# Functional common gamma chain is not required for mast cell proliferation and survival

**DOI:** 10.1186/1710-1492-8-S1-A22

**Published:** 2012-11-02

**Authors:** H Chapdelaine, D Canioni, G De Saint-Basile, O Hermine, A Fischer

**Affiliations:** 1 Department of Hematology, Hôpital Necker-Enfants Malades, Assistance Publique-Hôpitaux de Paris, Paris, France, 75743; 2 Centre de Référence Déficits Immunitaires Héréditaires, Hôpital Necker-Enfants Malades, France; 3 Department of Pathology, Hôpital Necker-Enfants Malades, France; 4 Centre d'Etudes des Déficits Immunitaires, Hôpital Necker-Enfants Malades, France; 5 Department of Pediatric Immuno-Hematology, Hôpital Necker-Enfants Malades, France

## Background

IL-15 is involved in the development and homeostasis of CD8 lymphocytes, NK and iNKT cells, and intraepithelial lymphocytes. It also promotes migration, proliferation and survival of mast cells. Its receptor on lymphocytes is a heterotrimeric complex which shares the IL2-Rβ and common gamma ( γ_c_ ) chains. IL-15-mediated signaling in mast cells might make use of an alternative receptor provisionally designated as IL-15RX. In a murine model, γ_c_-dependent signaling was shown to be essential for IL-4– and IL-9–induced proliferation and survival of mast cells, but not IL-15 even in the wild-type mouse.

## Methods

Skin biopsies of BCGitis lesions from 6 X-SCID patients were reviewed. Immunostaining using anti-CD117 ( 1/400, DAKO lab ) and tryptase ( 1/400, DAKO lab ) antibodies was performed. A similar BCGitis lesion from a patient with RAG SCID was tested with the same 2 antibodies as controls.

## Results

Mast cells were present in the skin biopsies of all patients presenting with X-SCID ; their number was not decreased compared to normal skin biopsies. The number of mast cells in the skin biopsy of the RAG SCID patient was also normal.

## Conclusion

These results suggest that, in humans, γ_c_ is not required for mast cell proliferation and survival. Its impact on IL-15-dependent migration and the possible role of IL-15RX warrant further studies.

